# Identification of Pediatric Oral Health Core Competencies through Interprofessional Education and Practice

**DOI:** 10.1155/2015/360523

**Published:** 2015-01-12

**Authors:** D. Hallas, J. B. Fernandez, N. G. Herman, A. Moursi

**Affiliations:** ^1^New York University College of Nursing (NYUCN), 433 First Avenue, New York City, NY 10010, USA; ^2^New York University College of Dentistry (NYUCD), 345 East 24th Street, New York City, NY 10010, USA

## Abstract

Over the past seven years, the Department of Pediatric Dentistry at New York University College of Dentistry (NYUCD) and the Advanced Practice: Pediatrics and the Pediatric Nurse Practitioner (PNP) program at New York University College of Nursing (NYUCN) have engaged in a program of formal educational activities with the specific goals of advancing interprofessional education, evidence-based practice, and interprofessional strategies to improve the oral-systemic health of infants and young children. Mentoring interprofessional students in all health care professions to collaboratively assess, analyze, and care-manage patients demands that faculty reflect on current practices and determine ways to enhance the curriculum to include evidence-based scholarly activities, opportunities for interprofessional education and practice, and interprofessional socialization. Through the processes of interprofessional education and practice, the pediatric nursing and dental faculty identified interprofessional performance and affective oral health core competencies for all dental and pediatric primary care providers. Students demonstrated achievement of interprofessional core competencies, after completing the interprofessional educational clinical practice activities at Head Start programs that included interprofessional evidence-based collaborative practice, case analyses, and presentations with scholarly discussions that explored ways to improve the oral health of diverse pediatric populations. The goal of improving the oral health of all children begins with interprofessional education that lays the foundations for interprofessional practice.

## 1. Interprofessional Education and Practice: Identification of Pediatric Oral Health Core Competencies

In 2005, New York University created a unique partnership between the College of Dentistry (NYUCD) and the College of Nursing (NYUCN) to foster interprofessional education through collaborative curriculum designs and practice environments with the goal of creating men and women of science whose education and practices were embedded in the principles of evidence-based dentistry, nursing, and medicine. Seven years ago, the pediatric faculty in their respective departments of dentistry and graduate pediatric nursing collaborated to provide interprofessional educational experiences in practice, research, and scholarship for second-year dental students, pediatric dental residents, and PNP students. The purpose of these collaborations was to provide formal interprofessional opportunities for these students to collaboratively examine the tenets of interprofessional practice and the evidence-based, culturally sensitive approaches to oral-systemic health care for culturally diverse, underserved children under 5 years old who are at high risk for dental caries. Two major goals were established by the nursing and dental faculty: (1) identify interprofessional pediatric oral health core competencies to enable the students to collaboratively plan interventions that reduce the incidence of early childhood caries (ECC); (2) promote evidence-based practice through interprofessional case analysis, discussions, and presentations. The purpose of this paper is to describe the design, implementation, and evaluation of dental and nursing interprofessional educational activities designed to accomplish these goals.

## 2. Background: Interprofessional Education (IPE) and Oral-Systemic Health Care

IPE is defined as “the collaborative process by which teams of health professions develop curricula and courses to coordinate and plan practical experiences jointly and team-teach groups of interdisciplinary [interprofessional] health professional students to provide holistic care through the life span” [1, p. 213]. The World Health Organization (WHO) commissioned a study group to analyze the effectiveness of 50 years of international experiences with IPE [2]. The outcome of their analysis produced the work entitled* Framework for Action on Interprofessional Education and Collaborative Practice* [3] that purported two significant WHO findings: (1) IPE fosters collaborative practice and (2) IPE improves health care outcomes.

The Interprofessional Education Collaborative (IPEC) was launched in 2012 with six founding member organizations leading the initiative to educate health care professionals as teams with a focus on patient-centered care [4]. Founding members included the American Dental Association (ADA), American Association of Colleges of Nursing (AACN), American Association of Colleges of Osteopathic Medicine (AACOM), American Association Colleges of Pharmacy (AACP), Association of Medical Colleges, and Association of Schools of Public Health. One major goal of IPEC was to formalize collaborative work started in 2009 that led to the report on “Core Competencies for Interprofessional Education and Practice” [4]. The purpose of the document was to articulate individual-level core competencies essential for health professionals to provide integrated, high-quality care [4]. The major tenets of IPEC included the following core competencies: (1) values and ethics for interprofessional practice; (2) roles and responsibilities for collaborative practice; (3) interprofessional communication; (4) interprofessional teamwork and team-based care. An open discussion paper by members of the Institute of Medicine [5] expounding upon the core principles and values of team-based health care used the following definition for team-based health care.

 Team-based health care is the provision of health services to individuals, families, and/or their communities by at least two health providers who work collaboratively with patients and their caregivers—to the extent preferred by each patient—to accomplish shared goals within and across settings to achieve coordinated, high-quality care [5, p. 5].

The principles of the IPEC can be readily applied to oral-systemic health care for the pediatric population. Oral-systemic health is defined as the relationship between oral health and systemic or whole body health [6, 7]. The vision of the AAOSH is to improve interdisciplinary [interprofessional] health care and collaboration by “changing public and professional awareness of the mouth-body health links” (AAOSH).

Early childhood caries (ECC) is defined as the presence of one or more decayed, missing, or filled tooth surfaces in any primary tooth in a child under 71 months of age (American Academy of Pediatric Dentistry [AAPD], 2014 [8]). ECC is the most prevalent unmet health care need for children under 71 months old [9, 10]. Interprofessional health care providers practicing in a team-based approach in primary health care and dental settings have the potential to improve the oral-systemic health of the pediatric populations. The authors believe that interprofessional collaborations among pediatric dental students, residents, and PNP students have the potential to improve the overall oral-systemic health of infants and young children.

## 3. Methods

### 3.1. Study Design

The authors designed a nonexperimental descriptive study to observe and describe student interactions during interprofessional educational activities involving pediatric dental students, residents, and PNP students. Educational activities included interprofessional education, clinical practice, and professional socialization for this population of health care providers. The interprofessional clinical activities were designed to identify the evidence-based pediatric oral health core competencies for pediatric dental and PNP students.

### 3.2. Participants

Study participants for the interprofessional oral health educational clinical experiences included Pediatric Nurse Practitioner students in their second year of study in a three-year part-time program, second-year dental students, and pediatric dental residents.

Pediatric nurse practitioners (PNPs) are currently educated at the master level to provide primary care (PNP-PC) or acute care (PNP-AC) to children and adolescents. By 2015, educational preparation for nurse practitioners will be transitioning to doctoral education as entry into advanced practice [11].

Dental students at NYUCD enter a 4-year program that includes didactic and clinical educational experiences. In their second year of study, the dental students participate in a program called ICE, which is an acronym for the “Initial Clinical Experience.” In addition, the ICE program was also their first organized interprofessional educational experience.

The pediatric dental residents were in either their first or second year of the residency program. The program is a total of 2 years of graduate training in the care of infants, children, and adolescents after graduating from a 4-year dental program.

Infants and young children between the ages of 6 months and 60 months who were enrolled in Head Start programs throughout the five boroughs of New York City were the recipients of oral health assessments and dental care by the interprofessional teams of dental and PNP students.

### 3.3. Settings

The Head Start programs throughout the five boroughs of New York City were the clinical practice sites for the ICE program for the dental and PNP students. The NYUCD pediatric clinic was also a clinical site for interprofessional oral health assessments, collaborations, and case discussions, among the dental residents, PNP students, and dental and nursing faculty.

### 3.4. Educational and Clinical Activities: The ICE Program

The Initial Clinical Experience (ICE) course was first developed in 2007, to introduce the dental student to pediatric dentistry and to pediatric caries prevention for children in diverse community settings. It provided dental students with a positive first experience in the role of the health professional. The dental students had the opportunity to clinically examine large numbers of children by performing oral health assessments and preventive treatments, including fluoride varnish to reduce the incidence of dental cavities in young children. The dental students completed a 6-week clinical rotation at various Head Start programs.

The ICE program was selected as the ideal program to introduce the PNP and dental students to interprofessional didactic and clinical experience in oral health care for at-risk children. The ICE program was refined to include one PNP student to rotate with eight dental students at various Head Start programs one day a week for a 6-week rotation.

Prior to their first clinical practice experience, the students completed the Smiles for Life program [12] and viewed a podcast created by the faculty on the basic principles of interprofessional practice. The students then met at NYUCD on the morning of their rotation and traveled together in a van to the clinical site. During the drive to the site, the faculty reviewed the day's assignment and facilitated a discussion on interprofessional roles and communication. At the end of each 6-week rotation, the dental and PNP students and at least one faculty member met during a lunch meeting and discussed the 6-week interprofessional experiences focusing on collaborative roles, ethics, communication, teamwork, and cultural implications for oral health care for diverse populations.

### 3.5. Use of Technology to Immediately Evaluate Student Performance

During the ICE program, each student was videotaped using an iPad at least two times during the 6-week rotation while performing an oral health assessment and fluoride varnish application. The faculty individually reviewed with each student the videotaped performance on the day of the clinical experience to offer the opportunity to the student to immediately recognize both the strengths of the assessment and areas for improvement.

### 3.6. Evaluation of Educational Activities

After completing the 6-week rotation in the ICE program, the PNP students took a 10-item written quiz in which the passing grade was set at 80%. If the students did not achieve the passing score or wanted to achieve a higher score, the students had the opportunity to take another quiz on the same topic to show improvement in the knowledge base or to achieve 100% on the quiz.

In addition, the PNP students demonstrated their oral health core competencies via an oral health simulation laboratory experience in which each student was presented with a case scenario and then performed an oral health assessment, fluoride varnish, and referrals, as needed, and provided anticipatory guidance to the “Standardized Parent” for the child.

Exam questions for the dental students were included in the semester final examination. Since 325 dental students participated in this experience over the course of the semester, their core competencies were evaluated using the iPad technology videotape and then by their respective dental faculty.

### 3.7. Educational and Clinical Activities: Collaborative Case Studies to Cultivate Student Teaching, Learning, and Leadership Skills

Students in all health care professions spend much of their time presenting cases to clinical faculty, although the case presentation format and dynamics have been minimally studied [13, 14]. The purpose of case presentations is quite simple: imparting information about patients to peers, faculty, clinicians, and consultants. Interprofessional case presentations coordinated by the students studying in various health professions presented to interprofessional faculty also serve as a vehicle for professional socialization [15–17]. Interprofessional case studies are also a vehicle for knowledge sharing among the professions and demonstrate a team-based approach for coordination of simple and complex care management and treatment plans [18]. In this collaborative format, in which PNP students were paired with pediatric dental residents, the case presentation served as a teaching/learning tool and a means to model interprofessional collaborations among faculty and students. These case presentations were exemplars of ways to improve clinical outcomes for the patient through an interprofessional team-based approach to care management.

Socialization among interprofessional students and faculty is a significant part of establishing the foundation for interprofessional team-based care after graduation. Socialization was fostered by scheduling about 15 minutes before the interprofessional collaborative case presentation for the students and faculty from each profession to meet and socialize in a collegial environment. After the case presentation, students and faculty again had about 15 minutes to socialize.

### 3.8. Data Collection

The following data were collected: the number of PNP and dental students who participated in the ICE program over a two-year, two-semester timeframe; the number of children in the Head Start program that received oral health assessments and fluoride varnish applications by the dental and PNP students; oral health assessments videotaped of each student in the ICE program using iPad technology; the number of students who performed collaborative case presentations and the number of students and faculty who attended the presentations; faculty evaluation of all case presentations; the results of the oral health 10-item assessment quiz administered to the PNP students at the end of the 6-week rotation; pass/fail results for PNP students after completion of the oral health simulation laboratory experience; pass/fail results on the oral health assessments and application of fluoride varnish by the dental students in the ICE program.

## 4. Results

Thirty PNP students, 325 dental students, and 18 residents participated in the program that was completed over a 2-year, 2-semester timeframe. In the first year of the program, 1,642 children in Head Start programs throughout the five boroughs of New York City received oral health assessments and fluoride varnish applications. If a parent was present, the parent received oral health anticipatory guidance. If the parent was not present, a letter was sent home to the parents advising the parents of the child's oral health status and whether further treatment was needed. In the second year of the program, 2,254 children received the same treatments. On average 30 children received oral health assessments and applications of fluoride varnish each day at various Head Start programs. Each student performed approximately five (5) oral health assessments and applications of fluoride varnish each day during their clinical rotation.

Each year, two dental residents and three PNP students presented two comprehensive collaborative case studies to dental students in the pediatric rotation, the pediatric residents, and faculty. Lunch was provided to the students and faculty to encourage interprofessional socialization. Fifty dental students, 18 residents, and 15 dental and nursing faculty and deans attended each presentation. Scholarly interactive discussions among students and faculty followed each presentation.

All 30 PNP students achieved at least the 80% passing score on the first attempt on the 10-item oral health quiz. Fifteen students received 100% on the first attempt; 10 students received 90%; and 5 received 80%. The 15 students who received less than 100% all took the second 10-item quiz and all but one student achieved a score of 100%.

All 30 PNP students participated in the simulation experience. All PNP students were able to demonstrate appropriate oral health assessments, application of fluoride varnish, and appropriate oral health anticipatory guidance based on the presented simulation.

All 325 dental students passed the ICE course and demonstrated competencies in oral health assessments and fluoride varnish application.

The faculty-student socialization at the end of the 6-week ICE rotation led to a number of student reflections concerning interprofessional clinical practice (Table 1). All students had the opportunity to reflect on their individual practices, interprofessional practice, and future collaborations. The students expressed interest in continuing collaborations to enhance their interprofessional experiences.

### 4.1. Core Competencies: Pediatric Nurse Practitioners and Pediatric Dentists

Core competencies for PNP and dental students in the area of oral health were identified through faculty analysis of students' performances during the ICE experiences using the iPad videotape.  Table 2 lists the identified interprofessional performance oral health core competencies for pediatric providers.

From the documentation of the faculty-student luncheon meetings, another essential interprofessional core competency emerged: oral-systemic health-illness cultural competencies that affect professional attitudes to effectively change population behaviors that promote wellness.

## 5. Recommendations

### 5.1. Incorporating Oral Health Core Competencies in Nurse Practitioner Curriculum

**Figure 1:**
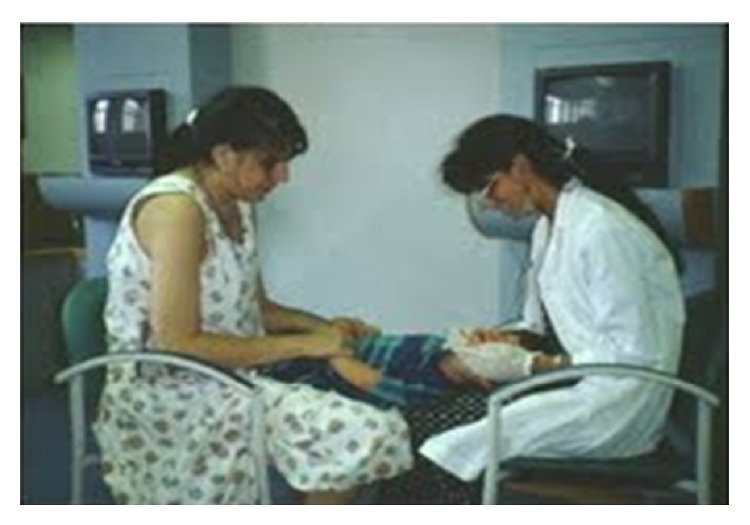
Knee-to-knee examination for infants and toddlers.

Primary care office based assessments are an essential part of the interprofessional initiative to reduce the incidence of early childhood caries (ECC). In addition to the interprofessional oral health performance and affective core competencies (see Tables 2 and 3) all NP students and, in fact, all pediatric providers, caring for children, need to learn how to perform a knee-to-knee examination and to incorporate this examination into their daily practices (see Figure 1). The Smiles for Life program [12] provides free access on how to perform oral health assessments on all age individuals, including specific video instructions, and performance of the knee-to-knee examination. Course models in the oral health education program relevant to pediatric practice include the following: Course #1: the relationship of oral to systemic health; Course #2: child oral health; Course #5: oral health and pregnancy; Course #6: caries risk assessment, fluoride varnish, and counseling; and Course #7: the oral examination. The Smiles for Life program was added as a required assignment for all of our NP and dental students (see Table 4 for oral educational health resources).

### 5.2. Recommended Curriculum Changes for Pediatric Dental Residents and General Dental Students

New interprofessional oral health core competencies for general dental students and pediatric dental residents need to focus on acquisition of knowledge, skills, and attitudes to care for infants and toddlers. New knowledge included oral-systemic health maintenance and anticipatory guidance for parents of infants and toddlers. Establishing a dental home by the infants' first birthday requires that dental students and residents be knowledgeable about infant nutrition and breast feeding guidelines including multivitamin intake for breast feeding mothers and for formula-fed infants. The dental students and residents must be aware of ever-changing trends in feeding infants and young children and how to advise parents about such trends based on scientific evidence. The recent trend by parents to feed infants and toddlers goats milk or raw milk presents challenges to all providers. Dental providers need to keep abreast of these trends and be prepared to discuss the potential oral-systemic consequences of such trends.

Managing the anxious child is a critical skill for general dental students who plan to practice as family dental provider. In addition, communication techniques to reassure parents of young children about the significance of oral health care are an essential skill. Pediatric dental office personnel and dentists will need to provide parent education in “one voice” with pediatric primary health care providers so that parents hear one consistent message about oral health care at each dental visit and at each pediatric health maintenance visit.

## 6. Discussion and Conclusions

This nonexperimental descriptive study to observe and describe interprofessional educational activities for pediatric dental students, residents, and PNP students has led to the identification of interprofessional performance and affective oral health core competencies for dental and pediatric primary health care providers. Inclusion of these core competencies as part of the curriculum in general dentistry programs and in pediatric and family nurse practitioner programs has the potential to improve the oral health care outcomes for at-risk infants and young children. Therefore, nurse practitioner and dental faculty need to enhance their current curriculum to enable students to be educated in interprofessional classrooms and to practice side by side in health care environments that provide opportunities to attain these oral health core competencies.

The ICE program provided the dental and PNP students with a positive first experience in interprofessional education and clinical practice and provided them with an opportunity to clinically perform oral health assessments on large numbers of infants, toddlers, and preschoolers. Caring for diverse populations of children requires health care professionals to be passionate about delivering comprehensive care that is culturally sensitive and focused on ways to educate parents to modify practices that negatively impact the oral-systemic health and well-being of infants and children. Faculty are responsible for cultivating reciprocal professional referrals among dentists who perform restorative procedures and pediatric primary care providers who not only refer children and parents for the procedures but also support parents by informing them of the need to improve oral-systemic health in their children. Behavior management for toddlers, preschoolers, and school-age children is another essential core competency that needs to be acquired by dental students, pediatric dental residents, and pediatric nurse practitioners. Gaining the cooperation of the child during primary care and dental office visits reduces the fears displayed by children in unfamiliar environments.

The use of the iPad videotapes proved to be valuable to each individual student. The student-faculty review showed the student immediate corrections that were needed to improve the performance of the oral health skills. The student then had the opportunity at the next clinical experience to perform the skill again and view the corrections.

The interprofessional socialization and discussion at the end of the 6-week ICE program and the case presentations provided unique opportunities for the dental and PNP students to collaboratively manage both simple and complex oral health care issues. Consistent with the findings of Thistlethwaite et al. [14], the students felt that this interprofessional education experience had the potential for increasing understanding among interprofessional health professionals. Identification and practice of interprofessional core competencies were enlightening for the students. The appealing premise of interprofessional education and clinical practice early in the students' educational experiences is that once health care professionals begin to work together in a collaborative manner, patient outcomes will improve.

It has been 13 years since the compelling report Oral Health in America by the Surgeon General which identified the poor oral health status of children from lower socioeconomic and diverse populations [19]. Implementation of interprofessional team-based oral health care to improve the oral-systemic health of our nation's infants and young children is long overdue.

## Figures and Tables

**Table 1:** Student reflections on interprofessional experiences.

*General dental students reflections *
(i) PNPs were helpful in calming the anxious child
(ii) PNPs were able to demonstrate ways to work with crying children
(iii) It gave us an opportunity to work with other professionals
(iv) I showed them what caries looked like
(v) I enjoyed working with the PNPs. They did not have the mindset of doing the dental work and leaving: they were much more engaged
with the children
*PNP students reflections *
(i) I was surprised to see the amount of children who had dental caries. The importance of educating parents in our practices and
collaborating with dental professionals is evident
(ii) Opportunities for collaboration include effective behavior modification strategies and reducing the child's anxiety and fears
(iii) Anticipatory guidance is so needed
(a) The parent of an overweight child who would not cooperate in the dental clinic offered to take the child to McDonalds
(b) The parent of an underweight child who would not cooperate offered to give the child a sticker

**Table 2:** Interprofessional performance oral health core competencies.

(i) Oral health assessments for infants, children, and adolescents	
(ii) Evaluation of primary and secondary dentition	
(iii) Recognition of malocclusions	
(iv) Identification of white spots	
(v) Treatment and management plans for white spots	
(vi) Identification of dental caries	
(vii) Nondental providers referrals to dental providers for treatment of dental caries	
(viii) Nondental providers referrals to dental providers for establishment of the dental home by the first birthday	
(ix) Dental injuries: identification, management, and referrals	
(x) Oral health care needs of children with special needs	
(xi) Application of fluoride varnish	

**Table 3:** Interprofessional affective oral health core competencies.

(i) Core competencies in the psychoemotional development of infants, toddlers, and preschool-age children
(ii) Oral-systemic health-illness cultural competencies
(iii) Appropriate chair side behavior management of toddlers and preschoolers
(iv) Parental education and anticipatory guidance
(a) Prevention of vertical transmission of oral organisms (*Streptococcus mutans*) from parent/caregiver to infants and children
(b) Pacifiers and current practice guidelines
(c) Recommendations for brushing and oral hygiene
(d) Related nutritional education including bottle and breast feeding
(e) Healthy nutrition guidelines for infants and children
(1) Avoiding foods that stick to the teeth including bananas and raisins
(f) Timing and preparation for establishing a dental home

**Table 4:** Oral health educational resources.

(i) American Academy of Pediatrics (AAP)	http://www.aap.org
(a) Online CE course	
(b) http://www2.aap.org/OralHealth/pact/index-cme.cfm	
(ii) American Academy of Pediatric Dentistry (AAPD)	http://www.AAPD.org
(iii) A Head Start on Oral Health: An Interactive Education Course for Head Start Employees of New York State	http://www.nypartnersinoralhealth.com
(a) Sponsored by NYUCD and New York State Dental Foundation Administered by NY State Attorney General's Office	
(iv) National Maternal & Child Oral Health Resource Center: Georgetown University	http://www.mchoralhealth.org
(v) Smiles for Life Oral Health Education Program (2010)	http://www.smilesforlifeoralhealth.com
(vi) Oral Health Nursing Education and Practice (OHNEP)	http://www.ohnep.org
